# Detection of Hepatitis E Virus in Shellfish Harvesting Areas from Galicia (Northwestern Spain)

**DOI:** 10.3390/v11070618

**Published:** 2019-07-05

**Authors:** Enrique Rivadulla, Miguel F. Varela, João R. Mesquita, Maria S.J. Nascimento, Jesús L. Romalde

**Affiliations:** 1 Departamento de Microbiología y Parasitología, CIBUS-Facultad de Biología, Universidade de Santiago de Compostela, 15782 Santiago de Compostela, Spain; 2 Instituto de Ciências Biomédicas Abel Salazar (ICBAS), Universidade do Porto, 4050-313 Porto, Portugal; 3 Epidemiology Research Unit (EPIUnit), Instituto de Saúde Pública da Universidade do Porto, 4050-600 Porto, Portugal; 4 Laboratório de Microbiologia, Departamento de Ciências Biológicas, Faculdade de Farmácia, Universidade do Porto, 4050-313 Porto, Portugal

**Keywords:** hepatitis E virus, shellfish, detection, genotyping, food safety

## Abstract

The hepatitis E virus (HEV) affects almost 20 million individuals annually, causing approximately 3.3 million acute liver injuries, 56,600 deaths, and huge healthcare-associated economic losses. Shellfish produced close to urban and livestock areas can bioaccumulate this virus and transmit it to the human population. The aim of this study was to evaluate the presence of HEV in molluscan shellfish, in order to deepen the knowledge about HEV prevalence in Galicia (northwestern Spain), and to investigate this as a possible route of HEV transmission to humans. A total of 168 shellfish samples was obtained from two different Galician rías (Ría de Ares-Betanzos and Ría de Vigo). The samples were analyzed by reverse transcription-quantitative PCR (RT-qPCR). RT-nested PCR and sequencing were used for further genotyping and phylogenetic analysis of positive samples. HEV was detected in 41 (24.4%) samples, at quantification levels ranging from non-quantifiable (<10^2^ copies of the RNA genome (RNAc)/g tissue) to 1.1 × 10^5^ RNAc/g tissue. Phylogenetic analysis based on the open reading frame (ORF)2 region showed that all sequenced isolates belonged to genotype 3, and were closely related to strains of sub-genotype e, which is of swine origin. The obtained results demonstrate a significant prevalence of HEV in bivalve molluscs from Galician rías, reinforcing the hypothesis that shellfish may be a potential route for HEV transmission to humans.

## 1. Introduction

The hepatitis E virus (HEV) was first documented in a patient with enterically transmitted non-A, non-B hepatitis in 1989 [1]. Nowadays, in addition to hepatitis A, B, C, and D, HEV is one of the major human hepatotropic viruses found around the world. According to the World Health Organization (WHO), HEV affects almost 20 million individuals annually, causing approximately 3.3 million acute liver injuries and 56,600 deaths, with large subsequent healthcare-associated economic losses [2].

HEV belongs to the family Hepeviridae within the genus *Orthopevirus*, which includes five genotypes that infect humans (HEV1, 2, 3, 4, and 7) [3,4]. HEV is a non-enveloped, positive-sense, single-stranded RNA virus, with an icosahedral capsid and a diameter between 27 and 34 nm [5,6]. The RNA genome of HEV is about 7.2 kb and is composed of three open reading frames (ORFs) [7]. ORF1 codes for a nonstructural polyprotein that is essential for viral RNA replication and infectivity. However, it is still debated whether this polyprotein functions as a single multifunctional polyprotein or undergoes proteolytic cleavage to produce individual active proteins [8]. ORF2 encodes a capsid protein responsible for virion assembly. ORF3 encodes a small protein involved in virion morphogenesis and release [9].

Genotypes 1 and 2 are prevalent in developing countries, including countries in Asia, Africa, and Central America [10,11]. Both genotypes are mainly restricted to humans and are transmitted by the consumption of fecal-contaminated water in areas with poor sanitation [12]. On the other hand, genotypes 3 and 4 have been confirmed as the main causes of zoonotic HEV worldwide; these genotypes represent the main reservoirs found in pigs and wild boars [4,10]. HEV7 has been detected in dromedary camels and in an immunocompromised person who regularly consumed camel meat and milk [3].

Over the last few years, the overall number of reported HEV cases has increased in Europe [13]. Although it is possible for infected humans to infect other people, person-to-person contact is not considered an efficient route of transmission. Other sources of exposure and transmission such as contaminated water, contaminated food (especially of swine origin), or contact with infected animals are more effective routes of transmission [3,10]. The millions of pigs raised in Europe produce huge amounts of manure and it is expected that spillover of such waste may contaminate the environment with HEV [3,14]. Shellfish harvesting areas contain variable amounts of viruses which form a part of the microbial plankton. However, other viruses that are excreted by infected people and animals (even when they are asymptomatic) have also been found in these shellfish harvesting areas, especially when these harvesting areas are located near urban or livestock areas [15,16].

The requirements of shellfish as a protein source has promoted the development of molluscan culture worldwide. Galicia (northwestern Spain) has a particular coastline topography characterized by the presence of fiord-like inlets, called rías, which are especially important as shellfish growing areas. In fact, Galicia is one of the most important regions of mussel production in the world, where they are cultured on floating rafts. Molluscan feeding behavior facilitates the bioaccumulation of pathogens, including viruses [17,18]. In addition, a long viral persistence has been demonstrated in these marine animals [18,19]. These two facts make molluscs important vectors for enteric diseases, especially when consumed raw or lightly cooked [20,21]. The periodic outbreaks of enteric viral diseases transmitted by molluscs have contributed to important economic losses by the seafood industry [22].

In a previous work by our team [23], HEV was detected in Galician mussels harvested from a location with low mussel production that was close to the city of A Coruña, one of the highest population density areas in Galicia (approximately 250,000 inhabitants), suggesting that those viruses were most likely of human origin. The aim of the present study was to perform a systematic surveillance of HEV prevalence in other harvesting areas that have a higher commercial interest and a lower urban influence, in order to confirm the previous results and hypothesis.

## 2. Materials and Methods

### 2.1. Shellfish Sampling

**Figure 1:**
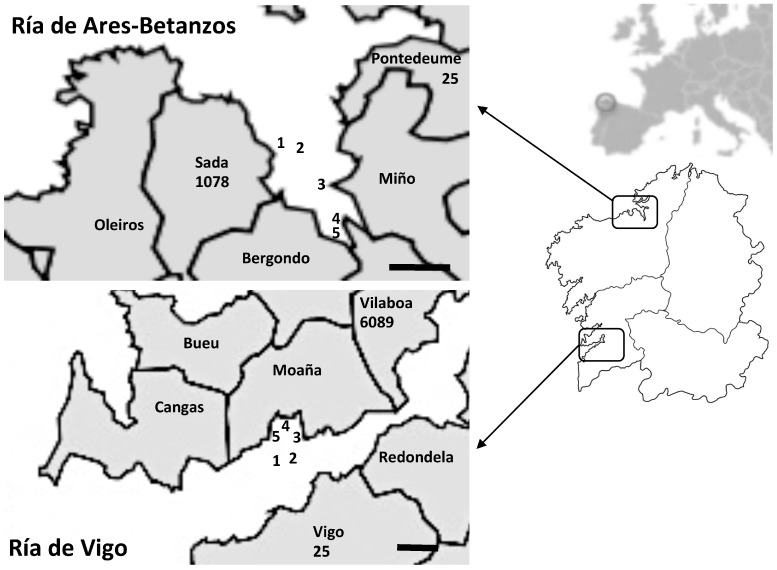
Shellfish sampling points analyzed in Ría de Ares-Betanzos (north) and Ría de Vigo (south). The number of officially registered pigs is indicated under the name of municipalities located near the harvesting areas (data from https://mediorural.xunta.gal/institucional/estatisticas/medio_rural/gando_porcino/). Scale bar, 2 km.

A total of 168 samples obtained from two different Galician rías (Ría de Ares-Betanzos and Ría de Vigo) (Figure 1), one in the north of Galician and the other in the south, were analyzed. These samples included wild- and raft-cultured Mediterranean mussels (*Mytilus galloprovincialis*), Manila and carpet-shell clams (*Ruditapes philippinarum* and *R. decussata*, respectively), and cockles (*Cerastoderma edule*) collected from different harvesting areas. Sampling points were classified as B areas (230–4600 MPN *Escherichia coli* per 100 g shellfish) or C (4600-46,000 MPN *Escherichia coli* per 100 g shellfish) according to European legislation [24]. Shellfish samples were collected monthly for 18 months, from January 2011 to June 2012, and were previously analyzed for other enteric viruses [25,26,27]. The original homogenates prepared from the digestive tissues of 10 mussels or 20 clams or cockles were stored at –80 °C, and were analyzed here for HEV detection.

### 2.2. Viral Recovery and RNA Extraction

Viral recovery from shellfish homogenates (2 g) samples was carried out following the ISO 15216-1:2017 specifications, with slight modifications as previously described [28,29]. Briefly, known amounts of Mengovirus clone vMC0 were spiked into each sample homogenate (10 µL, 10^3^ plaque forming units [PFU]) as a control for the RNA extraction efficiency [30]. After adding one volume of 0.1% peptone water pH 7.5 (1:1 w/v), homogenates were strongly shaken for one hour at 4 °C, centrifuged at 1000× *g* for 5 min, following which the supernatant was recovered. Viral RNA was extracted in duplicate from each homogenate using a Nucleospin^®^ RNA Virus Kit (Macherey-Nagel; Germany), from a sample volume of 150 μL according to the manufacturer’s protocol. The RNA was eluted in RNase-free sterile water and stored at −80 °C.

### 2.3. RT-qPCR Assay for HEV Screening and Quantification

A reverse transcription-quantitative PCR (RT-qPCR) assay targeting the ORF3 region of HEV [31] was applied to undiluted and diluted (1/10) RNA extracts. Negative controls containing no nucleic acid as well as positive controls (viral RNA) were included in each run. RT-qPCR was performed using an iTaq Universal PROBES One-Step Kit (Bio-Rad, Hercules, California, USA) with the primers JVHEVF/JVHEVR and the probe JVHEVP, a TaqMan^®^ probe containing a 5’6-carboxy fluorescein fluorophore and 3´ black hole quencher. The thermal cycling conditions were 42 °C for 5 min and 95 °C for 5 min, followed by 40 cycles of 95 °C for 3 s and 60 °C for 20 s. Extraction and amplification efficiencies were calculated according to the ISO 15216-1:2017 using Mengovirus and appropriate external controls (quantified HEV RNA from a clinical sample) [30,32].

Quantification was also carried out following the principles outlined in the ISO 15216-1:2017 as previously described [28,29]. Briefly, standard curves were constructed using serial dilutions of HEV RNA purified from a clinical sample (kindly donated by Dr. A. Aguilera from the University Hospital of Santiago de Compostela, Spain), in which the number of genome copies was plotted against the Ct values. Results were expressed as the number of RNA viral genome copies per gram of digestive tissue.

### 2.4. Broad-Spectrum Nested RT-PCR Assay

Viral RNA from all positive samples detected was subjected to genotyping using a RT nested-PCR protocol designed by Erker et al. [33] with minor modifications. The protocol amplifies the ORF2 region of the HEV genome. Briefly, 5 µL of viral RNA was added to 9.5 µL of the RT mixture from a RevertAid Reverse Transcriptase Kit (Thermo Scientific, USA) with the con-a1 (final concentration 1 µM) primer, in a final volume of 20 µL. The RT reaction was carried out at 42 °C for 60 min.

Next, cDNA samples were amplified by a nested PCR. In brief, for the first-round PCR 20 μL of synthesized cDNA, 0.5 µL of each of the con-a1 and con-s1 primers (final concentration 1 µM) and 4 µL sterile RNAse-free water were added to illustra^TM^ PuReTaq Ready-To-Go PCR Beads (GE Healthcare, Buckinghamshire, UK). The PCR conditions were as follows: a denaturation and activation step at 94 °C for 1 min, followed by 35 cycles of 94 °C for 20 s, 55 °C for 30 s, and 72 °C for 30 s, with a final extension at 72 °C for 10 min.

A 5 μL volume from the first-round PCR product was used as a template for the second-round PCR by mixing it with 0.5 µL of each of the con-a2 and con-s2 primers (final concentration 1 µM) and 19 µL sterile RNAse-free water, and adding it to illustra^TM^ PuReTaq Ready-To-Go PCR Beads (GE Healthcare, Buckinghamshire, UK). The PCR conditions were as follows: a denaturation and activation step at 94 °C for 5 min, followed by 35 cycles of 94 °C for 20 s, 55 °C for 30 s, and 72 °C for 40 s, with a final extension at 72 °C for 10 min.

The nested-PCR products were visualized on 2% agarose electrophoresis gel under UV light. Products of the expected length (145 bp) were purified and directly sequenced at STAB Vida Lda. (Portugal). Sequences obtained were processed with the Lasergene 7 software package (DNASTAR Inc., Madison, WI) and were aligned using the MEGA version 7 software package [34]. A phylogenetic tree was built by the maximum-likelihood method with a bootstrap analysis of 1000 replicates. Sequences of HEV reference strains were obtained from GenBank. Sequences of HEV strains detected in the present study are available at GenBank under accessions LR215969 to LR215972.

## 3. Results and Discussion

Consumption of shellfish has been identified and linked as a risk factor for HEV transmission in Asian countries including China [35], Japan [36,37], Korea [38], Thailand [39], and Vietnam [40]. However, in Europe the route of HEV transmission has not clearly been established. Whilst some investigations point to the absence of HEV in shellfish [41,42,43], other studies have documented the presence of HEV in wild and commercially available mussels of Italy [44,45], the Netherlands [46], Spain [23,47], and the UK [48,49]. Regardless, few cases of hepatitis E linked to shellfish consumption in Europe have been described so far [40,50].

As mentioned, previous studies carried out by our team detected HEV in Galician mussels from a harvesting area that has a high human influence with prevalence of 14.8% [23]. In the present study, HEV was detected in a higher percentage of the shellfish (24.4%), despite the harvesting areas being located further from urban settlements. Detection in the north estuary Ría de Ares-Betanzos (18 positive samples) was slightly lower than in the southern estuary Ría de Vigo (23 positive samples), yielding prevalence values of 20.0% and 29.4%, respectively. In an attempt to determine the possible origin of such contaminations, and taking into account the negligible urban waste that could arrive to both areas, the presence of porcine livestock around the harvesting areas was analyzed. According to official information (https://mediorural.xunta.gal/institucional/estatisticas/medio_rural/gando_porcino/), 32.8% of Spanish pig farms are located in Galicia, with 28,401 pigs registered. A total of 1103 pigs are bred in pig farms around Ría de Ares-Betanzos, whereas 6114 animals are bred near Ría de Vigo close to the shellfish sampling points (Figure 1). These data could explain not only the higher prevalence in the southern ría, but also indicate a possible swine origin of the HEV detected.

Differences in contamination were also observed among the diverse bivalve species, although without statistical significance (Fisher’s exact test; *p* > 0.05). Thus, 20 out of 70 (28.5%) samples of cultured mussels were HEV positive, as well as 13 out of 35 (37.1%) of wild mussel samples. Clams and cockles showed lower prevalences with five out of 31 (16.1%) and three out of 32 (9.3%) positive samples, respectively. Such differences may be due to the different location of the sampling points and the influence of currents or other hydrographic variables, although further studies are needed to confirm these hypotheses. No clear seasonal pattern could be observed, with HEV being detected throughout the studied period (Table 1) with few exceptions, such as the absence of positive samples during February and March for both years in the north estuary. This finding may be explained by the high persistence of the virus in the environment [51] or by a continued supply of virus coming from nearby pig farms.

The observed prevalence of HEV in the Galician shellfish samples was considerably higher than those reported in other studies in European countries [44,45,46,47,49], with the exception of that by Crossan et al. [48], who observed HEV at a prevalence of up to 92% in shellfish from a harvesting area in Scotland located near a slaughterhouse and a meat preparation purification plant that processes pigs. On the other hand, quantification levels in our study ranged from non-quantifiable (<10^2^ copies of the RNA genome (RNAc)/g tissue) to 1.1 × 10^5^ RNAc/g tissue detected in a cultured mussel sample from the north estuary. Thus, eight samples (19.5%) showed viral levels below the limit of quantification, one sample of cultured mussel from the southern estuary rendered 4.0 × 10^2^ RNAc/g tissue, and the other 31 positive samples (75.6%) showed quantifications ranging from 10^3^ to 10^4^ RNAc/g tissue, which are in line with the values obtained by Crossan et al. [48].

**Figure 2:**
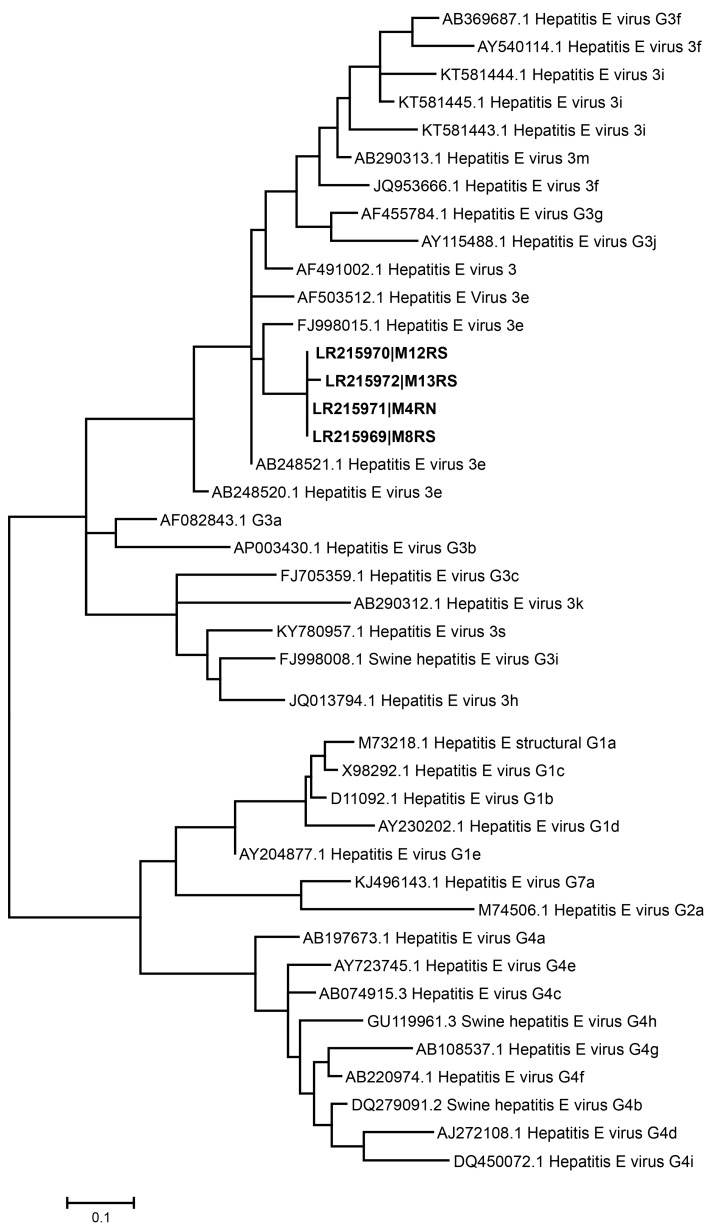
Phylogenetic tree of hepatitis E virus (HEV) samples, based on sequences of the ORF2 region, constructed by maximum likelihood analysis using MEGA 7. GenBank accession numbers of the shellfish (in bold) and reference strains used are detailed in the tree. Scale bar, 0.1 nucleotide substitutions.

Phylogenetic analysis based on the ORF2 region showed a high similarity (>96%) among the four HEV sequences obtained from Galician shellfish, regardless of their geographic origin. All sequences corresponded to genotype 3, clustering in the phylogenetic tree with sequences of the sub-genotype e (Figure 2). Sequences from Galician shellfish were closely related to strains from swine and wild boar of different geographical origins, with similarities in the analyzed amplicons higher than 93.5% [52,53,54]. Unfortunately, to our knowledge no HEV surveys have yet been performed on porcine livestock in Galicia in order to experimentally confirm the link between shellfish and swine sequences; thus, further studies are needed to uncover this aspect. The same genotype had been previously reported for shellfish from other areas in Galicia [23], as well as in shellfish and water from production areas in Italy [43].

The joint analysis of the results of the present study with those of previous work from our laboratory using the same samples [25,26,27] revealed the presence of mixed contaminations with other enteric viruses. Thus, of 41 HEV positive samples, 34 (83%) showed the presence of more than one virus, and only seven (17%) samples were positive exclusively for HEV. Of these 34 samples, 10 (24%) were also positive for norovirus (NoV) GI, seven (17%) for NoV GII, seven (17%) for sapovirus (SaV), and two (5%) for Aichi virus (AiV). The other eight (19%) samples showed the co-presence of HEV with two or more viruses (Table 1).

With the exception of the inner most sampling points of the north estuary, which were classified as C areas according to the EU regulations [24], the remaining shellfish samples were obtained from B areas and therefore were considered suitable for human consumption after a depuration process. However, it is important to mention that depuration processes are not completely effective for viral removal [55] and that the viral levels detected in some of the shellfish samples were similar to the theoretical infectious dose reported elsewhere [56]. These facts suggest that shellfish may be a potential route for HEV transmission to humans.

## 4. Conclusions

The obtained results demonstrated a significant prevalence of HEV in bivalve molluscs from harvesting areas in Galicia and, contrary to previous investigations, an important level of mixed contaminations with other enteric viruses that could strengthen the severity of possible foodborne outbreaks. The evidence obtained in the present work reinforces the idea that pig farms have a strong influence on the appearance of HEV contamination, especially in Ría de Vigo. Although further studies are needed in order to confirm such a hypothesis and to accurately determine the significance of HEV detection in shellfish, the data obtained could be helpful to refine the risk assessment of foodborne transmission of HEV, and will support the establishment of appropriate measures to reduce the risk of shellfish-associated illnesses.

## Figures and Tables

**Table 1:** Detection of HEV (◎) along the study period in each harvesting area indicating the present of mixed contaminations with other enteric viruses. Data for norovirus genotype I (◼), norovirus genotype II (◆), Aichi virus (**❁**), Sapovirus (*), and hepatitis A virus (**★**) were obtained from previous works [25,26,27].

Zone	Sample ^1^	2011													2012					
		Jan	Feb	Mar	Apr	May	Jun	Jul	Aug	Sep	Oct	Nov	Dec		Jan	Feb	Mar	Apr	May	Jun
Northe	RC mussels (1)				◎◼							◎**❁**	◎**❁**					◎		
	RC mussels (2)	◎◆					◎◼				◎◆							◎*	◎◆**★**	
	W mussels (3)	◎			◎◼					◎◼		◎◆	◎◆**❁**					◎◆*		
	Clams (4)								◎◼	◎◆										
	Cockles (5)											◎◼◆*								
South	RC mussels (1)			◎◼	NT			◎◼	◎◼	◎◼◆						◎				◎◆
	RC mussels (2)	◎◼			NT					◎◆			◎◆		◎*				◎◼◆	
	W mussels (3)				NT		◎	◎	◎◼				◎*		◎*					◎◆**★**
	Clams (4)				NT						NT	◎*	◎*		NT		◎	NT	NT	◎◆**★**
	Cockles (5)				NT			◎					◎*		NT			NT	NT	

^1^ RC mussels, raft cultured mussels; W mussels, wild mussels; Nt, not tested. The correspondent sampling point is reported in brackets.

## References

[1] Wang Y., Zhao C., Qi Y., Geng Y., Wang Y. (2016). Hepatitis E Virus. Hepatitis E Virus (Advances in Experimental Medicine and Biology Series 948).

[2] World Health Organization (WHO) Hepatitis E. https://www.who.int/en/news-room/fact-sheets/detail/hepatitis-e.

[3] Kenney S.P. (2019). The current host range of hepatitis E viruses. Viruses.

[4] Smith D.B., Purdy M.A., Simmonds P. (2013). Genetic variability and the classification of hepatitis E virus. J. Virol..

[5] Balayan M.S., Usmanov R.K., Zamyatina D.I., Karas F.R. (1990). Brief report: Experimental hepatitis E infection in domestic pigs. J. Med. Virol..

[6] Reyes G.R., Purdy M.A., Kim J.P., Luk K.C., Young L.M., Fry K.E., Bradley D.W. (1991). Hepatitis E virus (HEV): The novel agent responsible for enterically transmitted non-A, non-B hepatitis. Gastroenterol. Jpn..

[7] Meng X.J., Anderson D.A., Arankalle V.A., Emerson S.U., Harrison T.J., Jameel S., Okamoto H., King A.M.Q., Adams M.J., Carstens E.B., Lefkowitz E.J. (2012). Hepeviridae. Virus Taxonomy: Classification and Nomenclature of Viruses: Ninth Report of the International Committee on Taxonomy of Viruses.

[8] Parvez M.K. (2017). The hepatitis E virus nonstructural polyprotein. Future Microbiol..

[9] Kamar N., Abravanel F., Lhomme S., Rostaing L., Izopet J. (2015). Hepatitis E virus: Chronic infection, extra-hepatic manifestations, and treatment. Clin. Res. Hepatol. Gastroenterol..

[10] Khuroo M.S., Khuroo M.S., Khuroo N.S. (2016). Hepatitis E: Discovery, global impact, control and cure. World J. Gastroenterol..

[11] Siddiqui A.R., Jooma R.A., Smego R.A. (2005). Nosocomial outbreak of hepatitis E infection in Pakistan with possible parenteral transmission. Clin. Infect. Dis..

[12] Purcell R.H., Emerson S.U. (2008). Hepatitis E: An emerging awareness of an old disease. J. Hepatol..

[13] European Centre for Disease Prevention and Control (ECDC) (2017). Hepatitis E in the EU/EEA, 2005–2015.

[14] Lewis H.C., Wichmann O., Duizer E. (2010). Transmission routes and risk factors for autochthonous hepatitis E virus infection in Europe: A systematic review. Epidemiol. Infect..

[15] Wyn-Jones A.P., Sellwood J. (2001). Enteric viruses in the aquatic environment. J. Appl. Microbiol..

[16] Barreira D.M., Ferreira M.S., Fumian T.M., Checon R., de Sadovsky A.D., Leite J.P., Miagostovich M.P., Spano L.C. (2010). Viral load and genotypes of noroviruses in symptomatic and asymptomatic children in Southeastern Brazil. J. Clin. Virol..

[17] Loisy F., Atmar R.L., Le Saux J.C., Cohen J., Caprais M.P., Pommepuy M., Le Guyader F.S. (2005). Use of rotavirus virus-like particles as surrogates to evaluate virus persistence in shellfish. Appl. Environ. Microbiol..

[18] Schwab K.J., Neill F.H., Estes M.K., Metcalf T.G., Atmar R.L. (1998). Distribution of Norwalk virus within shellfish following bioaccumulation and subsequent depuration by detection using RT-PCR. J. Food Prot..

[19] Metcalf T.G., Melnick J.L., Estes M.K. (1995). Environmental virology: From detection of virus in sewage and water by isolation to identification by molecular biology—a trip of over 50 years. Annu. Rev. Microbiol..

[20] Cheng P.K., Wong D.K., Chung T.W., Lim W.W. (2005). Norovirus contamination found in oysters worldwide. J. Med. Virol..

[21] Bellou M., Kokkinos P., Vantarakis A. (2013). Shellfish-borne viral outbreaks: A systematic review. Food Environ. Virol..

[22] Pommepuy M., Le Guyader F.S., Le Saux J.C., Guilfoyle F., Doré B., Kershaw S., Lees D., Lowther J.A., Morgan O.C., Romalde J.L., Børresen T. (2008). Reducing microbial risk associated with shellfish in European countries. Improving Seafood Product for the Consumer.

[23] Mesquita J.R., Oliveira D., Rivadulla E., Abreu-Silva J., Varela M.F., Romalde J.L., Nascimento M.S. (2016). Hepatitis E virus genotype 3 in mussels (*Mytilus galloprovinciallis*), Spain. Food Microbiol..

[24] (2004). European Regulation (EC) N° 854/2004 of the European Parliament and of the Council of 29 April 2004 laying down specific rules for the organization of official controls on products of animal origin intended for human consumption. Off. J. Eur. Union.

[25] Manso C.F., Romalde J.L. (2013). Detection and characterization of hepatitis A virus and norovirus in mussels from Galicia (NW Spain). Food Environ. Virol..

[26] Polo D., Varela M.F., Romalde J.L. (2015). Detection and quantification of hepatitis A virus and norovirus in Spanish authorized shellfish harvesting areas. Int J. Food Microbiol.

[27] Varela M.F., Polo D., Romalde J.L. (2016). Prevalence and genetic diversity of human Sapovirus in shellfish from commercial production areas in Galicia, Spain. Appl. Environ. Microbiol..

[28] International Organization for Standardization (ISO) (2013). Microbiology of Food Chain—Horizontal Method for Determination of Hepatitis A Virus and Norovirus Using Real-Time RT-PCR—Part 1: Method for Quantification.

[29] Rivadulla E., Varela M.F., Romalde J.L. (2017). Low prevalence of Aichi virus in molluscan shellfish samples from Galicia (NW Spain). J. Appl Microbiol..

[30] Costafreda M.I., Bosch A., Pintó R.M. (2006). Development, evaluation, and standardization of a real-time TaqMan reverse transcription-PCR assay for quantification of hepatitis A virus in clinical and shellfish samples. Appl. Environ. Microbiol..

[31] Jothikumar N., Cromeans T.L., Robertson B.H., Meng X.J., Hill V.R. (2006). A broadly reactive one-step real-time RT-PCR assay for rapid and sensitive detection of hepatitis E virus. J. Virol. Methods.

[32] Pintó R.M., Costafreda M.I., Bosch A. (2009). Risk assessment in shellfish-borne outbreaks of hepatitis A. Appl. Environ. Microbiol..

[33] Erker J.C., Desai S.M., Schlauder G.G., Dawson G.J., Mushahwar I.K. (1999). A hepatitis E virus variant from the United States: Molecular characterization and transmission in cynomolgus macaques. J. Gen. Virol..

[34] Kumar S., Stecher G., Tamura K. (2016). MEGA7: Molecular evolutionary genetics analysis Version 7.0 for bigger datasets. Mol. Biol. Evol..

[35] Gao S., Li D., Zha E., Zhou T., Wang S., Yue X. (2015). Surveillance of hepatitis E virus contamination in shellfish in China. Int. J. Environ. Res. Public Health.

[36] Li T., Miyamura T., Takeda N. (2007). Short report: Detection of hepatitis E virus RNA from the bivalve yamato-shijimi (*Corbicula japonica*) in Japan. Am. J. Trop. Med. Hyg..

[37] Inagaki Y., Oshiro Y., Hasegawa N., Fukuda K., Abei M., Nishi M., Okamoto H., Ohkohchi N. (2015). Clinical features of hepatitis E virus infection in Ibaraki, Japan: Autochthonous hepatitis E and acute-on-chronic liver failure. J. Exp. Med..

[38] Song Y.J., Jeong H.J., Kim Y.J., Lee S.W., Lee J.B., Park S.Y., Song C.S., Park H.M., Choi I.S. (2010). Analysis of complete genome sequences of swine hepatitis E virus and possible risk factors for transmission of HEV to humans in Korea. J. Med. Virol..

[39] Namsai A., Louisirirotchanakul S., Wongchinda N., Siripanyaphinyo U., Virulhakul P., Puthavathana P., Myint K.S., Gannarong M., Ittapong R. (2011). Surveillance of hepatitis A and E viruses contamination in shellfish in Thailand. Lett. Appl. Microbiol..

[40] Koizumi Y., Isoda N., Sato Y., Iwaki T., Ono K., Ido K., Sugano K., Takahashi M., Nishizawa T., Okamoto H. (2004). Infection of a Japanese patient by genotype 4 hepatitis E virus while traveling in Vietnam. J. Clin. Microbiol..

[41] Grodzki M., Schaeffer J., Piquet J.C., Le Saux J.C., Chevé J., Ollivier J., Le Pendu J., Le Guyader F.S. (2014). Bioaccumulation efficiency, tissue distribution and environmental occurrence of hepatitis E virus in bivalve shellfish from France. Appl. Environ. Microbiol..

[42] La Rosa G., Fratini M., Spuri Vennarucci V., Guercio A., Purpari G., Muscillo M. (2012). GIV noroviruses and other enteric viruses in bivalves: A preliminary study. New Microbiol..

[43] Krog J.S., Larsen L.E., Schultz A.C. (2014). Enteric porcine viruses in farmed shellfish in Denmark. Int. J. Food Microbiol..

[44] La Rosa G., Proroga Y.T.R., De Medici D., Capuano F., Iaconelli M., Della Libera S., Suffredini E. (2018). First detection of hepatitis E virus in shellfish and in seawater from production areas in Southern Italy. Food Environ. Virol..

[45] Purpari G., Macaluso G., Di Bella S., Gucciardi F., Mira F., Di Marco P., Lastra A., Petersen E., La Rosa G., Guercio A. (2019). Molecular characterization of human enteric viruses in food, water samples, and surface swabs in Sicily. Int. J. Infect. Dis..

[46] Pol-Hofstad I.E., Rutjes S.A., Gerssen A., Poelman M., van der Linden A.D. (2014). Evaluation of the sanitary status of the Dutch shellfish production waters over a 7 year period. Proceedings of the 9th International Conference on Molluscan Shellfish Safety.

[47] Diez-Valcarce M., Kokkinos P., Söderberg K., Bouwknegt M., Willems K., de Roda-Husman A.M., von Bonsdorff C.H., Bellou M., Hernández M., Maunula L. (2012). Occurrence of human enteric viruses in commercial mussels at retail level in three European countries. Food Environ. Virol..

[48] Crossan C., Baker P.J., Craft J., Takeuchi Y., Dalton H.R., Scobie L. (2012). Hepatitis E virus genotype 3 in shellfish, United Kingdom. Emerg. Infect. Dis..

[49] O’Hara Z., Crossan C., Craft J., Scobie L. (2018). First report of the presence of hepatitis E virus in Scottish-harvested shellfish purchased at retail level. Food Environ. Virol..

[50] Said B., Ijaz S., Kafatos G., Booth L., Thomas H.L., Walsh A., Ramsay M., Morgan D. (2009). Hepatitis E outbreak on cruise ship. Emerg. Infect. Dis..

[51] Johne R., Trojnar E., Filter M., Hofmann J. (2016). Thermal stability of hepatitis E virus estimated by a cell culture method. Appl. Environ. Microbiol..

[52] Adlhoch C., Wolf A., Meisel H., Kaiser M., Ellerbrok H., Pauli G. (2009). High HEV presence in four different wild boar populations in East and West Germany. Vet. Microbiol..

[53] Inoue J., Takahashi M., Ito K., Shimosegawa T., Okamoto H. (2006). Analysis of human and swine hepatitis E virus (HEV) isolates of genotype 3 in Japan that are only 81–83 % similar to reported HEV isolates of the same genotype over the entire genome. J. Gen. Virol..

[54] Clemente-Casares P., Pina S., Buti M., Jardi R., Martín M., Bofill-Mas S., Girones R. (2003). Hepatitis E virus epidemiology in industrialized countries. Emerg. Infect. Dis..

[55] Polo D., Feal X., Romalde J.L. (2015). Mathematical model for viral depuration kinetics in shellfish: A useful tool to estimate the risk for the consumers. Food Microbiol..

[56] Hewitt P.E., Ijaz S., Brailsford S.R., Brett R., Dicks S., Haywood B., Kennedy I.T., Kitchen A., Patel P., Poh J. (2014). Hepatitis E virus in blood components: A prevalence and transmission study in southeast England. Lancet.

